# Paediatric psoriasis and biologics: An evidence gap in plain sight

**DOI:** 10.1111/jdv.70309

**Published:** 2026-03-25

**Authors:** Stephan Traidl, Sascha Gerdes

**Affiliations:** ^1^ Department of Dermatology and Allergy Hannover Medical School Hannover Germany; ^2^ Department of Dermatology, Venereology and Allergology, Center for Inflammatory Skin Diseases University Hospital Schleswig‐Holstein, Campus Kiel Kiel Germany

Despite the broad armamentarium of effective biologic therapies available for the treatment of moderate‐to‐severe psoriasis in adults, and the recent initiation of discussions on potential disease modification or even curing,^1^ therapeutic options for children and adolescents remain comparatively limited.

Aljalfan et al. provide a rigorous synthesis of the available randomized controlled trials for biologic therapies in paediatric plaque psoriasis and highlights this persistent imbalance.^2^

Psoriasis is not uncommon in childhood. Population‐based studies estimate a prevalence of approximately 0.5%–1% among children and adolescents, with up to one third of patients experiencing disease onset before the age of 18.^3^, ^4^ Beyond visible skin involvement, paediatric psoriasis is associated with substantial physical and psychosocial burden, impaired quality of life and early trajectories of cardiometabolic and inflammatory comorbidities.^4^ Nevertheless, the therapeutic evidence base in this age group remains markedly narrower than that established for adult patients.

Against this background, the network meta‐analysis by Aljalfan et al. represents an important contribution. Across randomized controlled trials, biologic therapies demonstrated meaningful efficacy and acceptable short‐term safety in children and adolescents with moderate‐to‐severe plaque psoriasis. The analysis also highlights structural limitations, including small trial numbers, limited sample sizes and few direct comparisons, necessitating cautious interpretation of treatment rankings.

The findings also underscore a central discrepancy between adult and paediatric psoriasis care. In adults, multiple biologics targeting TNFα, IL‐17 and IL‐23 are approved and routinely used.^5^, ^6^ In contrast, paediatric approvals are confined to a limited subset of these agents and specific age categories, despite shared disease biology. Consequently, treatment decisions in children often rely on extrapolation from adult data and are still frequently constrained by slower regulatory timelines and restricted labelling.

In addition, paediatric‐specific guidance remains limited. Existing European recommendations acknowledge the usefulness of biologics in children and adolescents with moderate‐to‐severe disease when conventional systemic treatment is inadequate or inappropriate.^5^, ^6^ However, the guideline also highlights the limited paediatric trial data underpinning these recommendations. Updated guidelines are urgently needed—ideally aligning paediatric therapeutic goals more closely with those used in adults. Importantly, these goals should include, where feasible, complete or near‐complete skin clearance as a realistic treatment target. The findings of the present meta‐analysis underline that several already approved paediatric biologics can indeed support such ambitious outcomes.

Another relevant consideration is that, in many countries, systemically approved therapies for paediatric psoriasis consist almost exclusively of biologic agents and, more recently, a single small‐molecule therapy. While this may appear restrictive, it may also facilitate access to highly effective targeted treatments when systemic therapy is indicated.

Encouragingly, the paediatric development pipeline is expanding. In December 2025, the IL‐23 inhibitor guselkumab was approved for children aged 6 years and older in Europe. Additionally, multiple ongoing clinical trials are currently evaluating targeted therapies already approved in adults, particularly IL‐23 inhibitors such as tildrakizumab (NCT03997786) and risankizumab (NCT04435600), as well as other modern targeted therapies across established inflammatory pathways (e.g. deucravacitinib: NCT04772079, bimekizumab: NCT06425549). A notable example is icotrokinra, an oral IL‐23 receptor antagonist developed in parallel for adolescents and adults, reflecting a gradual shift towards earlier paediatric inclusion.

The present network meta‐analysis reinforces two key messages. First, biologic therapies constitute an effective and generally well‐tolerated treatment option for paediatric plaque psoriasis. Second, therapeutic decision‐making in this population continues to be shaped by incomplete comparative evidence, underscoring the importance of individualized care, shared decision‐making and long‐term pharmacovigilance.

In conclusion, the study by Aljalfan et al. provides an important benchmark for the current state of evidence in paediatric psoriasis. Future progress will depend on earlier inclusion of paediatric cohorts in development programmes, renewal and harmonization of paediatric‐specific guidelines and closer alignment of paediatric treatment targets—ideally including skin clearance—with adult standards. As the therapeutic landscape continues to broaden, network‐based analyses such as the present study will remain valuable in helping clinicians navigate an increasingly diverse set of effective treatment options and in supporting equitable, evidence‐based care for children and adolescents with psoriasis.

## CONFLICT OF INTEREST STATEMENT

Stephan Traidl has received honoraria for lectures or scientific from AbbVie, ALK, Almirall, Galderma, Janssen/JNJ, LaRoche‐Posay, LEO Pharma, Lilly, Novartis and Regeneron/Sanofi. Sascha Gerdes has been an advisor and/or received speakers' honoraria and/or received grants and/or participated in clinical trials for the following companies: AbbVie, Affibody AB, Akari Therapeutics Plc, Almirall‐Hermal, Amgen, Anaptys Bio, Argenx BV, AstraZeneca AB, Biogen Idec, Bioskin, Boehringer Ingelheim, Bristol Myers Squibb, Celgene, Dermira, Eli Lilly, Foamix, Forward Pharma, Galderma, Hexal AG, Incyte Inc., Janssen‐Cilag, Johnson & Johnson, Klinge Pharma, Kymab, Leo Pharma, Medac, MSD, Neubourg Skin Care GmbH, Novartis, Pfizer, Principia Biopharma, Regeneron Pharmaceuticals, Sandoz Biopharmaceuticals, Sanofi‐Aventis, Trevi Therapeutics and UCB Pharma.

## Data Availability

Data sharing not applicable to this article as no datasets were generated or analysed during the current study.

## References

[1] Lwin SM , Azrielant S , He J , Griffiths CEM . Curing psoriasis. J Invest Dermatol. 2024;144(12):2645–2649. 39436345 10.1016/j.jid.2024.09.012

[2] Aljalfan AA , Maniya MT , Sachedina D , Almuhaidib SR , Alharbi AS , Alamrie R , et al. Biologics for treatment of paediatric plaque psoriasis: a systematic review and network meta‐analysis. J Eur Acad Dermatol Venereol. 2026;40(4):597–609. 10.1111/jdv.70084 41090545

[3] Raychaudhuri SP , Gross J . A comparative study of pediatric onset psoriasis with adult onset psoriasis. Pediatr Dermatol. 2000;17(3):174–178. 10886746 10.1046/j.1525-1470.2000.01746.x

[4] Augustin M , Glaeske G , Radtke MA , Christophers E , Reich K , Schäfer I . Epidemiology and comorbidity of psoriasis in children. Br J Dermatol. 2010;162(3):633–636. 19922529 10.1111/j.1365-2133.2009.09593.x

[5] Nast A , Smith C , Spuls PI , Avila Valle G , Bata‐Csörgö Z , Boonen H , et al. EuroGuiDerm guideline on the systemic treatment of psoriasis vulgaris – part 1: treatment and monitoring recommendations. J Eur Acad Dermatol Venereol. 2020;34(11):2461–2498. 33349983 10.1111/jdv.16915

[6] Nast A , Smith C , Spuls PI , Avila Valle G , Bata‐Csörgö Z , Boonen H , et al. EuroGuiDerm guideline on the systemic treatment of psoriasis vulgaris – part 2: specific clinical and comorbid situations. J Eur Acad Dermatol Venereol. 2021;35(2):281–317. 33547728 10.1111/jdv.16926

